# Implementation of suicide bereavement support: a scoping review

**DOI:** 10.3389/fpubh.2024.1474641

**Published:** 2024-11-14

**Authors:** Tescha Nicholls, Karolina Krysinska, Lennart Reifels, Dzenana Kartal, Karl Andriessen

**Affiliations:** ^1^Centre for Mental Health and Community Wellbeing, Melbourne School of Population and Global Health, The University of Melbourne, Melbourne, VIC, Australia; ^2^Centre for Health Policy, Melbourne School of Population and Global Health, The University of Melbourne, Melbourne, VIC, Australia

**Keywords:** bereavement, implementation, scoping review, postvention, suicide

## Abstract

**Introduction:**

Approximately one in five people will experience a death by suicide during their lifetime. Struggling to understand their experiences, people bereaved by suicide often require formal support including support groups, online resources and discussion forums. While previous research has focused on examining experiences of grief, little is known about the implementation of support programs for people bereaved by suicide.

**Methods:**

Adhering to PRISMA-ScR guidelines, eight databases were searched for peer-reviewed studies that focused on the strategies or evaluation of the implementation of suicide postvention programs.

**Results:**

Sixteen studies met the eligibility criteria. A narrative synthesis of study findings mapped to the Proctor implementation framework shows that a variety of implementation outcomes have been assessed by different studies; however, there is limited use of formal implementation frameworks to guide analysis.

**Discussion:**

Recommendations from this review indicate the need for consistent terminology and better utilisation of implementation frameworks to guide postvention research and provide a well-rounded view of implementation. Increasing the use of reliable and validated tools to measure implementation outcomes will also increase the rigour of implementation research in this field.

**Systematic review registration:**

https://doi.org/10.17605/OSF.IO/4RB92.

## 1 Introduction

The World Health Organization reports that more than 700,000 people die by suicide each year, making it a significant public health priority (1). Suicide is also the fourth leading cause of death among 15–29-year-olds (1).

Suicide not only impacts the individual but can have profound and lifelong impacts on those left behind (1). A meta-analysis of population-based data estimated that one in five people will experience a death by suicide in their lifetime (2). Cerel et al. (3) considered the impact of suicide on a broad scale from mere exposure to deeply affected and estimated that up to 135 individuals could be affected by one suicide. Experiencing the suicide of a close person is a potentially devastating event, putting the bereaved individual at risk of adverse grief, poor mental health and suicidal behaviour (4). People bereaved by suicide often struggle with “why” questions and meaning making, and they often receive less social support compared to other bereaved individuals (5).

Given the devastating impact of suicide bereavement, formal support is often warranted, especially for those closest to the deceased individual. This may include support groups, counselling, online resources or discussion forms. Support that is designed specifically to aid recovery and prevent the development of adverse effects for people bereaved by suicide is termed “postvention” (6). Effective delivery of suicide postvention support is critical for this population due to their needs, particularly when considered the perceived intentionality and preventability of the death, as well as the perceived stigma and trauma associated with suicide (7).

Postvention research over recent decades has mostly focused on examining suicide grief experiences (8), with an increasing interest in the effectiveness of postvention programs, as reported in several systematic reviews (8–10). Abbate et al. (9) also addressed the acceptability of postvention programs. However, while each of these reviews identified methodological limitations of the included studies, and provided recommendations for future research, none specifically investigated the implementation of postvention programs, which is the aim of this scoping review.

Implementation science aims to bridge the gap between research and practice by promoting the systematic uptake of evidence-based practice and research findings to improve healthcare (11, 12). Implementation science can also help identify effective strategies to improve the likelihood of successful implementation (13).

Despite considerable advances in the broader field of implementation science, it is still not prevalent in the suicide prevention field (14). Interventions aimed at addressing high rates of suicide are often complex and delivered in response to immediate population needs or motivated by policy directives or funding opportunities (14). Due to the ethical, methodological, and practical challenges with evaluating suicide prevention interventions, implementation-focused research largely relies on observational studies (15). Strengthening the focus on implementation science within the field will help ensure that effective interventions can be implemented appropriately, rather than assuming their impactful translation into practice (14). Although Reifels et al. (14) and Krishnamoorthy et al. (15) speak directly to suicide prevention, similar sentiments seem to apply to evaluating the implementation of suicide postvention programs (16).

The research question for this scoping review is: What is currently known about the implementation of postvention programs for people bereaved by suicide? The objectives of this scoping review are to examine (a) how supports and programs for people bereaved by suicide have been implemented; (b) how implementation has been evaluated; and (c) which implementation outcomes have been evaluated.

## 2 Methods

This scoping review was guided by the methodological framework developed by Arksey and O’Malley (17) and the Preferred Reporting Items for Systematic Reviews and Meta-analyses Extension for Scoping Review (PRISMA-ScR) guidelines (18). The protocol for this scoping review was registered at Open Science Framework on 26th October 2023 and updated 14th February 2024 (19).

### 2.1 Eligibility criteria

Studies were included if: (i) the population of interest was people bereaved by suicide (any age), and/or providers of suicide bereavement support, (ii) the study focused on the strategies and/or evaluation of the implementation of suicide postvention programs, (iii) the study design was qualitative or quantitative, and (iv) the study was published in a peer-reviewed journal in English.

Studies were excluded if: (i) the study was a systematic review, commentary, opinion article or a letter, and (ii) if the full text was unavailable.

The inclusion criteria were intentionally broad in order to capture a range of study designs that investigate implementation of postvention programs (i.e., providing support for people bereaved by suicide). The study populations included both people bereaved by suicide and providers of suicide postvention support programs to meet the aim of the review (i.e., to review what is known of the implementation of postvention programs, how it has been evaluated, and which outcomes have been evaluated).

### 2.2 Search strategy

The search was conducted in January 2024 in eight databases: Medline OVID, EBM Reviews, Embase, Emcare, PsycINFO, CINAHL, Scopus, and Web of Science. The citation list of each of the included papers, as well as of systematic reviews of postvention research, were searched for relevant studies. A forward citation search was also conducted. The searches were not limited by date of publication or location.

**Figure 1:**
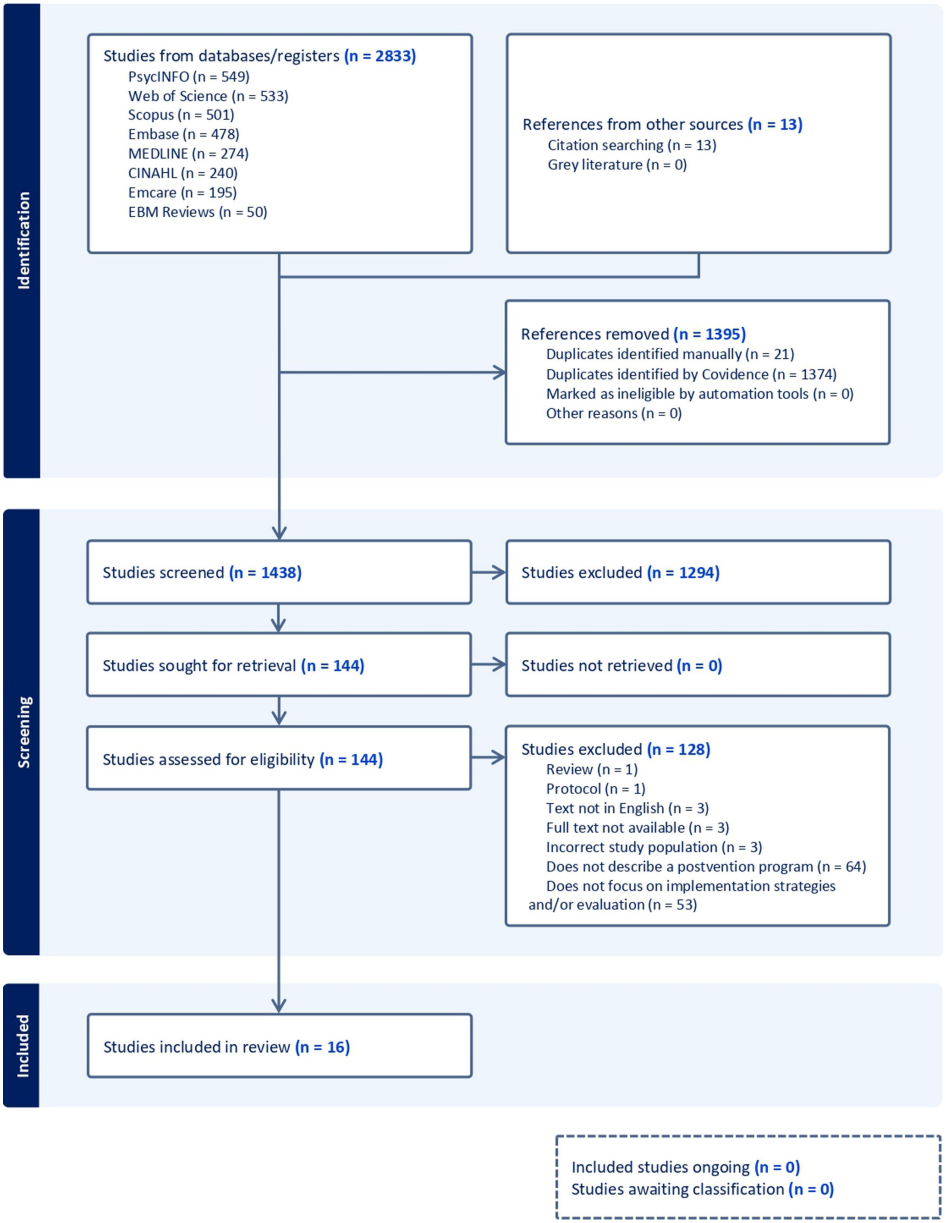
PRISMA flow diagram. Adapted from Tricco et al. (18).

The research team developed the search string, with advice from a University of Melbourne librarian, consisting of four key concepts: suicide, bereavement, postvention programs, and implementation science. Supplementary material S1 includes the detailed search string. Figure 1 presents the search and selection process.

### 2.3 Data selection and extraction

All articles identified in the searches were imported into the COVIDENCE systematic review software platform for screening. TN and KK independently undertook a two-step screening process: (1) title and abstract screening, and (2) full text screening. We aimed for inter-rater agreement above 75% for each screening step (20). The initial trial of 20% of eligible references for title and abstract screening received an inter-rater agreement of 91% and all conflicts were quickly resolved through discussion between TN, KK, and KA. TN screened the remaining titles and abstracts.

TN and KK independently screened the full text articles and reached an inter-rater agreement of 79%. KA screened all articles with conflicting votes and arrived at a final decision. All reasons for exclusion were recorded and reported in Figure 1.

Data were extracted into an Excel table seeking standard bibliographic information in addition to the type of study, the study population, bereavement information, postvention program type, implementation outcome(s) of interest, outcome measures, and summary results of implementation or evaluation. TN and KA independently extracted data from two articles and compared results. As there were only minor differences in the amount of information extracted from the main results section, TN completed data extraction and KK served as a second rater.

### 2.4 Data synthesis

The implementation outcomes framework reported in Proctor et al. (21) was used to assess, map and synthesise the findings. The framework consists of eight key implementation outcomes that can serve as indicators of success during the implementation process (21). The implementation outcomes are (1) *acceptability* (measures whether the intervention itself is satisfactory, palatable or agreeable), (2) *adoption* (measures the uptake or action to employ an intervention), (3) *appropriateness* (measures the suitability, perceived fit or relevance of the intervention), (4) *feasibility* (measures the actual fit or the extent to which the intervention can be carried out in the setting), (5) *fidelity* (measures if the intervention was delivered as described), (6) *implementation cost* (measures all costs associated with implementation), (7) *penetration* (measures the level of integration into the service setting), and (8) *sustainability* (measures whether the intervention is incorporated into ongoing practice) (21). The authors acknowledged that these concepts can sometimes overlap or be conflated in the literature, however, they stressed the importance of considering each of these outcomes separately to fully evaluate the implementation of interventions (21).

To guide the synthesis of the results presented here, each study outcome was assessed against Proctors implementation outcomes, using the definitions provided above. To do this, we foremostly utilised the authors’ stated implementation outcome when available. When the implementation outcomes were not clearly defined, or aligned with any implementation framework, TN and DK assessed each study outcome against Proctor et al. (21) definition of implementation outcomes.

## 3 Results

### 3.1 Study population

Our search identified 16 eligible studies that were published between 1992 and 2023, 11 of which were published more than ten years ago (see Table 1). Five of the studies were conducted in the United States of America (22–26), four were conducted in Australia (27–30) and two were conducted in both Northern Ireland (31, 32) and Canada (33, 34). The remaining three studies were conducted in New Zealand (35), Taiwan (36) and Germany (37). The age range of participants ranged from 5 years old (31) to 95 years old (26) although not all studies stated the age range. All studies except one (34) had samples with greater numbers of females compared to males.

**Table 1:** Overview table of study characteristics including program types and implementation outcomes.

Authors	Date of publication	Title	Type of program	Implementation outcomes assessed
Barlow, CA; Schiff, JW; Chugh, U; Rawlinson, D; Hides, E; Leith, J. (33)	2010	An evaluation of a suicide bereavement peer support program.	General postvention program	Feasibility
				Appropriateness
				Acceptability
				Fidelity
Bowden, C. (35)	2011	Waves: A psycho-educational program for adults bereaved by suicide	General postvention program	Feasibility
				Appropriateness
				Acceptability
Braiden HJ; McCann M; Barry H; Lindsay C. (31)	2009	Piloting a therapeutic residential for children, young people and families bereaved through suicide in Northern Ireland	Postvention program for children	Appropriateness
				Acceptability
Callahan, J. (22)	1996	Negative effects of a school suicide postvention program--a case example.	School-based postvention program	Feasibility
				Fidelity
Clark, SE; Jones, HE; Quinn, K; Goldney, RD; Cooling, PJ. (27)	1993	A support group for people bereaved through suicide.	General postvention program	Feasibility
				Appropriateness
				Fidelity
Clements A; Nicholas A; Martin KE; Young S. (28)	2023	Towards an Evidence-Based Model of Workplace Postvention	Training program for providers of postvention support	Acceptability
Daigle, MS; Labelle, RJ. (34)	2012	Pilot evaluation of a group therapy program for children bereaved by suicide.	Postvention program for children	Appropriateness
				Adoption
Farberow, NL. (23)	1992	The Los Angeles Survivors-After-Suicide program: An evaluation	General postvention program	Feasibility
				Appropriateness
				Acceptability
Galway, K; Forbes, T; Mallon, S; Santin, O; Best, P; Neff, J; Leavey, G; Pitman, A. (32)	2019	Adapting Digital Social Prescribing for Suicide Bereavement Support: The Findings of a Consultation Exercise to Explore the Acceptability of Implementing Digital Social Prescribing within an Existing Postvention Service.	Internet-based postvention program	Feasibility
				Appropriateness
				Acceptability
Grossman, J; Hirsch, J; Goldenberg, D; Libby, S; Fendrich, M; Mackesy-Amiti, ME; Mazur, C; Chance, GH. (24)	1995	Strategies for school-based response to loss: Proactive training and postvention consultation.	Training program for providers of postvention support	Feasibility
				Acceptability
Hazell, P.; Lewin, T. (29)	1993	An evaluation of postvention following adolescent suicide	School-based postvention program	Feasibility
				Appropriateness
Hill, NTM; Walker, R; Andriessen, K; Bouras, H; Tan, SR; Amaratia, P; Woolard, A; Strauss, P; Perry, Y; Lin, A. (30)	2022	Reach and perceived effectiveness of a community-led active outreach postvention intervention for people bereaved by suicide.	General postvention program	Feasibility
				Appropriateness
Lu, YJ.; Chang, HJ.; Tung, YY.; Hsu, MC.; Lin, MF. (36)	2011	Alleviating psychological distress of suicide survivors: Evaluation of a volunteer care program	Training program for providers of postvention support	Appropriateness
Pfeffer CR; Jiang H; Kakuma T; Hwang J; Metsch M. (25)	2002	Group Intervention for Children Bereaved by the Suicide of a Relative	Postvention program for children	Feasibility
				Fidelity
Treml, J; Nagl, M; Linde, K; Kundiger, C; Peterhansel, C; Kersting, A. (37)	2021	Efficacy of an Internet-based cognitive-behavioural grief therapy for people bereaved by suicide: a randomized controlled trial.	Internet-based postvention program	Feasibility
				Appropriateness
				Acceptability
Zisook, S; Shear, M K; Reynolds, *CF*; Simon, NM; Mauro, C; Skritskaya, NA; Lebowitz, B; Wang, Y; Tal, I; Glorioso, D; Wetherell, JL; Iglewicz, A; Robinaugh, D; Qiu, X. (26)	2018	Treatment of Complicated Grief in Survivors of Suicide Loss: A HEAL Report.	Combined medication and therapy postvention program	Acceptability

Time since bereavement varied greatly between the studies. Callahan (22), Barlow et al. (33) and Hazell and Lewin (29) were conducted immediately following the suicide, within a few days or weeks. However, Barlow et al. (33) also included people bereaved by suicide more than 20 years ago. Farberow (23), Pfeffer et al. (25), Zisook et al. (26), Braiden et al. (31), and Treml et al. (37) had varying bereavement periods but were not considered as being in the immediate aftermath. Five studies did not specify the time since bereavement or (27, 30, 32, 34, 35). The remaining three study populations were providers of postvention support, and thus time since bereavement was not cited (24, 28, 36).

Each of the studies included unique kinship relationships to the deceased. Nine of the studies included a range of close kinship losses, including wife, husband, mother, father or siblings (23, 25–27, 30, 31, 33, 34, 37). Programs targeted towards children and adolescents included some discussion of the relationship to the deceased. School-based postvention programs commented on differences between students considered close to the deceased in comparison to students not considered at high risk of distress (22, 29). The postvention programs targeted towards children after the loss of a parent or sibling commented on the chance of profound lifelong impacts if they did not receive any bereavement support due to the close kinship relationship (25, 31, 34). The remaining programs targeted towards adults did not separate results according to the type of kinship loss, and this relationship does not appear to have explicitly affected implementation of the postvention program. Studies focused on providers of postvention support for adults did not identify kinship information (28, 32). Two studies did not provide any bereavement information (35, 36).

Two of the programs specifically targeted bereavement of a schoolmate and commented on differences identified between students who were known to be close to the deceased and those who were not considered at high risk of severe distress (22, 29). The programs for children involving the suicide of a parent or sibling identified that the close proximity had the capability of causes profound lifelong impacts without intervention (25, 31, 34). Other programs targeted towards adults did not explicitly separate results according to the type of relationships to the deceased and does not appear to have explicitly affected implementation of the programs.

### 3.2 Program characteristics

The studies evaluated a variety of postvention programs aimed at different populations and settings. Three studies assessed postvention programs developed specifically for children [aged five to 16 years (31), six to 12 years (34), and six to 15 years (25)], two studies assessed school-based postvention programs (22, 29), two assessed internet-based programs (32, 37). One study specifically evaluated medication and therapy focused postvention support for suicide-bereaved individuals suffering from complicated grief (26). Three studies assessed training programs for people providing postvention support (24, 28, 36). This included training school staff (24), funeral workers of suicide bereavement funerals (28), and volunteers providing postvention support (36). The remaining five studies assessed general postvention programs which were not specific to a particular population, type of program, or relationship to the deceased (23, 27, 30, 33, 35). Aside from the two internet-based programs, each of these studies assessed programs delivered face-to-face.

Six of the included studies were mixed methods evaluations (28, 30, 31, 33–35). These evaluations used surveys combined with interviews (28, 33), observation (34) or focus groups (35). Hill et al. (30) used data from the Australian Bureau of Statistics alongside interviews for evaluation. Three studies were quantitative evaluations (23, 24, 36), one was a qualitative evaluation (25), and one reported the results of a co-design workshop (32). Three of the included studies were case studies (22, 27, 29). Two studies explored the evaluation of suicide postvention programs solely through author commentary (26, 37), and the remaining two (24, 25) provided additional data related to implementation through author commentary.

### 3.3 Implementation outcomes

No study utilised an explicit conceptual implementation framework to guide their selection of implementation outcomes of interest. Five of the studies (26, 29, 30, 36, 37) explicitly utilised one of the implementation outcomes found within Proctor et al.’s (21) framework (e.g., acceptability or appropriateness). However, none of the studies specifically pre-defined these terms, nor were they explicitly related any conceptual framework. According to our analysis using Proctor et al. (21) framework, three studies explored a single implementation outcome (26, 28, 36), 12 studies explored two or three implementation outcomes (22–25, 27, 29–32, 34, 35, 37), and only one study explored more than three implementation outcomes (33).

All other studies assessed implementation outcomes via proxy measures only (i.e., according to their assessment/tools utility), for example, Daigle et al. (34) used the increased anger scores on the Beck Youth Inventories of Emotional and Social Impairment (34, 38) as a proxy for the appropriateness of the program.

Validated tools were used in three of the studies to assess a component of implementation. These tools were the Beck Youth Inventories of Emotional and Social Impairment (34, 38), Brief Symptoms Ratings Scale-5 (36, 39), and the Therapist Performance Scale (25, 40). Additionally, the Suicide Risk Index (29) was developed by the researchers but was determined to have concurrent validity in the study. Although some of these tools aim to measure features of effectiveness, they were also used as a proxy for an implementation measure. Other validated tools only related to effectiveness were used but are not discussed here. Implementation data were also gathered through interviews (25, 28, 30, 31, 33), focus groups (35), quantitative surveys (often using Likert scales and/or open-ended questions) (23, 28, 30, 31, 33–36), and discussion from the authors (22, 24, 26, 27, 29, 37). The remaining study (32) presented results from a co-design workshop. Where validated tools were not used, authors utilised made-for-purpose surveys or interview guides according to their implementation interests, which were not explicitly analysed for reliability, or they incorporated commonly used proxy measures [such as measures of drop-out rates to assess acceptability as seen in Treml et al. (37) and Zisook et al. (26)], to analyse a component of implementation.

### 3.4 Feasibility

The most common implementation outcome addressed by the included studies was feasibility. Twelve studies either evaluated or discussed a component of feasibility in their analysis (22–27, 29, 30, 32, 33, 35, 37).

Four of the studies (27, 32, 33, 35) identified structural components of postvention programs that increased the feasibility of implementing the intervention. Participants of postvention programs identified flexibility (33), additional support via e-mail or phone (33), simplicity of service (32) and timing of the program (35) as key components increasing the feasibility of each program. Providers of postvention support noted key facilitators of implementation were limiting administrative burden (30), proactively planning how to manage anger or a destructive atmosphere in peer support meetings (27) and retaining highly skilled facilitators to lead meetings that allow for balanced participation (27).

Key barriers to feasibility included difficulty finding suitable times and locations to meet due to time constraints of participants (33), additional responsibility and burden on school counsellors and social workers who “*began to feel that it was their responsibility to directly prevent additional suicides”* [(22), p. 136], the inability of school staff to accurately identify at-risk students in need of postvention support (29, 32) and the inability to offer support if consent for contact was not provided (32). Treml et al. (37) also identified that its internet-based model meant that it was “*more difficult to intervene in a crisis”* [(37), p. 11], although the authors noted that this did not render the program entirely infeasible. Gossman et al. (24) identified that staff turnover and confidentiality laws preventing the disclosure of witness names delayed postvention program entry into schools and meant program implementers were unable to provide additional support to higher risk students.

A key component of evaluating the feasibility of an intervention is identifying issues with poor recruitment or retention (21). Farberow (23) and Pfeffer et al. (25) specifically identified reasons for poor retention, which speaks to their feasibility. Farberow (23) received feedback from participants who dropped out that “*the location was too far away; [they] found help elsewhere; and in a few cases cost was given as a major factor*” [(23), p. 6]. Pfeffer et al. (25) identified the passive control model as the reasoning behind 75% of non-intervention families dropping out throughout the course of the program compared to just 18% of those receiving the intervention. Pfeffer et al. (25) also identified difficulty recruiting as the reason for updating the protocol (discussed in the fidelity section below), however, aside from identifying that of the 112 families contacted for participation, ten refused, and 27 could not be found, no specific reason for poor recruitment was provided (25). No other studies specifically evaluated recruitment methods, nor their effect on the feasibility of implementation.

Interestingly Zisook et al. (26) concluded that “*this study provides preliminary evidence that CGT [Complicated Grief Therapy] is feasible to administer…*” [(26), p. e6]. However, the evidence of such feasibility relies on the low dropout rates in the CGT component of the study, which the authors explicitly link to the acceptability of the intervention, rather than its feasibility. This will be discussed in the acceptability section below.

Some of the recommendations from participant evaluations addressed components of implementation that could aid in increasing the feasibility of the programs. Participants from Barlow et al. (33) requesting more flexibility in the training schedule for peer supporters. Bowden (35) acknowledged that there needs to be a considerable focus on the choice and training of facilitators as “*the program syllabus is great but without the right facilitators it could flop*” [(35), p. 29].

### 3.5 Appropriateness

Eleven studies evaluated a component of appropriateness (23, 27, 29–37). Seven of these studies explored aspects of the program that were seen to improve the appropriateness of the program being evaluated (23, 27, 31–33, 35, 37). Meetings in a “*safe*” [(31), p. 91; (35), p. 29], “*neutral*” [(33), p. 923], “*accessible… uplifting, and comfortably furnished”* [(27), p. 164] locations were key components that spoke to the appropriateness of the setting of the program. Galway et al. (32) described how the flexibility of the program allowed and encouraged follow-up at different times with the bereaved, as the initial point of contact may not have been an appropriate time to engage grieving individuals with support services. Several of the participants from the Peer Support Program (33) identified that “*matches [between the suicide-bereaved and peer supporters] based on similar losses were a powerful factor in their ability to connect with each other*” [(33), p. 924], speaking to the appropriateness of the program. In contrast, the participants of the Los Angeles Survivors-After-Suicide Program (23) identified that it had been “*highly advantageous*” [(23), p. 33] to have grouped different losses together as it allowed them to “*see how much effort had been made to help, as well as how much pain had been experienced by everyone involved*” [(23), p. 33].

A common component of appropriateness addressed by Clark et al. (27) and Farberow (23) was regarding the role of non-suicide-bereaved counsellors. Farberow (23) identified that “*most benefit was found by using a professional and a survivor as co-leaders. The two serve overlapping but very different and necessary functions*” [(23), p. 33]. Similarly, Clark et al. (27) identified that it was “*helpful for a counselor who had not been bereaved through suicide to work with the group at meetings to provide objectivity and direction and to back up the support workers*” [(27), p. 16]. None of the remaining articles specifically addressed the appropriateness of including both suicide-bereaved and non-suicide-bereaved counsellors in the program.

While there were several positive aspects of the programs described by the authors to increase the appropriateness of the interventions, there were also negative aspects that could hinder the overall appropriateness of implementation. Increased anger scores identified using the Beck Youth Inventories of Emotional and Social Impairment (38) were thought to be due to a “*pace unsuited to all children”* [(34), p. 355], negatively impacting the overall appropriateness of the program to help children manage their grief. The selection criteria used by the program evaluated by Hazell and Lewin (29) may not have been entirely appropriate given “*proximity to completed suicide alone was a relatively weak predictor for subsequent suicidal ideation and behaviour. The style and focus of counselling may itself have been inappropriate”* [(29), p. 108]. The online structure of the program evaluated by Treml et al. (37) contributed to the overall appropriateness of the intervention given that “*the therapist’s feedback could be misinterpreted, or the therapist could draw the wrong conclusions”* [(37), p. 11].

The focus of the evaluation by Lu et al. (36) was to understand if it was appropriate for novice volunteers to provide support to suicide-bereaved individuals. Results from the evaluation determined “*four novice group suicide survivors [with existing moderate to severe distress] increased Brief Symptoms Rating Scale-5 scores in the third month, indicating worsening distress*” [(36), p. 453]. These results speak directly to the appropriateness of novice volunteers providing support for suicide-bereaved individuals with moderate or severe distress compared to those with low distress.

Recommendations from the evaluations also addressed components of appropriateness. Clark et al. (27) identified the need for the venue of the program to not to be connected to governments or other institutions that may further stigmatise people seeking support. Lu et al. (36) recommended the inclusion of routine evaluation of participants to monitor distress to provide additional support where needed. As mentioned earlier, Hazell and Lewin (29) identified concerns with the appropriateness of selection criteria for students to receive counselling. Recommendations to overcome this issue included “*invit[ing] students to select themselves for postvention counselling… [or] a brief by systematic screening of all students for the presence of risk factors for suicide*” [(29), p. 108]. Although the authors acknowledged these strategies also have limitations, they may be able to identify more students in need of counselling (29).

### 3.6 Acceptability

Nine of the included studies evaluated a component of acceptability (23, 24, 26, 28, 31–33, 35, 37). Acceptability of the implementation of the interventions was most commonly analysed using Likert-scale questionnaires which asked questions about how helpful, useful, worthwhile, favourable, beneficial or satisfactory programs were. Results from these surveys were largely positive [e.g., 4.4 out of 5 average helpfulness score for participants of the Peer Support Program (33), parents of children participating in the Barnardo’s Child Bereavement Service rated helpfulness 9 out of 10 (35), 96.9% of participants rated the training for suicide bereavement funerals as somewhat or very useful (28) and 92% of participants of the Los Angeles Survivors-After-Suicide Program rated their experience favourably (23)]. Program length was one area of acceptability to receive more conflicting results from surveys. More than 50% of participants of the school-based postvention program evaluated by Grossman et al. (24) wanted more training, and 51% of participants of the Los Angeles Survivors-After-Suicide Program thought there were too few sessions [although 41% felt it was *“just right”* (23), p. 32].

The focus group discussions that evaluated the Waves program (35) identified numerous aspects of the program that addressed the acceptability of the program. The structure of the program whereby they created a “*community of grievers*” was a particularly important component that spoke to the acceptability of the program [(35), p. 7]. “*It is good to be part of the community and be with people who have had similar experiences*” [(35), p. 7] and that “*it’s not just me – I’m not alone*” [(35), p. 7]. They also noted the importance of the suicide-bereaved facilitator as “*it helps if the facilitators have had an experience of suicide in their lives to share and recall*” [(35), p. 7]. These comments from participants highlighted how the structure and content speak to the acceptability of the implementation of the intervention.

Increasing the acceptability of a program requires overcoming barriers to engagement (21). Three studies explicitly explored how their program was able to overcome particular barriers (30, 32, 37). The online models used by Galway et al. (32) and Treml et al. (37) were able to address similar barriers. Treml et al. (37) identified potential barriers as the “*fear of being judged or stigmatized…. distance/unavailability, as well as a lack of information, time or financial resources*” [(37), p. 3]. Their internet-based model “*offer[ed] more geographic and time flexibility and anonymity as well as faster attainability*” [(37), p. 3], helping to overcome some of the aforementioned barriers. Galway et al. (32) identified that their online model was able to overcome similar barriers, aided by the anonymity and accessibility of internet services. The specific barriers identified by Hill et al. (30) included a “*lack of awareness of services, distance, cost and waitlist times*” [(30), p. 9]. They found that using the active Primary Care Navigator model, “*barriers to help seeking [were] assessed during initial contact with bereaved individuals to facilitate better access to postvention services*” [(30), p. 9], helping the address the overall acceptability of the program.

Despite Proctor et al. (21) usually associating retention rates with feasibility, two of the included studies (26, 37) specifically discussed low dropout rates as a proxy for high acceptability. Treml et al. (37) stated that “*considering the low dropout rate in this study, the ICBGT [internet-based cognitive-behavioural grief therapy] seems to be also highly accepted*” [(37), p. 11]. Similarly, Zisook et al. (26) used retention rates as a measure of acceptability of the therapy intervention to respond to suicide bereavement. “*CGT [complicated grief therapy] completion rates were high and comparable across all bereavement categories, indicating that CGT is an acceptable treatment approach for suicide survivors with CG [complicated grief]*” [(37), p. e5]. The authors here have explicitly identified that high retention is a sign of high acceptability of the intervention.

Some of the evaluations specifically asked for recommendations for the program in the future. In terms of acceptability, two studies (24, 35) requested more training time, while others (29, 30, 33) requested more follow-up sessions to provide on-going support. Three studies (23, 27, 31) suggested changes to the content and structure of the program itself. This included more structured learning (more informational materials and practical advice) (23), guest speakers and reading lists (27), and more chances for non-directive therapeutic sessions (31). The authors suggested that incorporating these recommendations could increase the acceptability of the programs moving forward.

### 3.7 Fidelity

Four of the included studies addressed a component of fidelity (22, 25, 27, 33) with three of these (22, 25, 27) making changes to the protocol throughout the implementation process. These were required due to practical reasons, or to respond to the needs of suicide-bereaved participants.

Practical reasons included Pfeffer et al. (25) experiencing poor recruitment and therefore the authors assigned families in an alternating pattern to the intervention or control group rather than using random assortment given the “*need to avoid delay in starting the intervention*” [(25), p. 512].

Responding to the needs of the bereaved participants prompted changes to two studies (22, 27). Callahan (22) found that the counselling sessions with distressed students which lasted for long periods of time (1–2 h) “*seemed to stir up more emotional intensity that was resolved*” [(22), p. 112–113] and that meeting with students in groups “*seemed to lead to an increased intensity of melodramatic and romanticized feelings*” [(22), p. 112]. Moving forward, authors recommended only seeing students by appointment, suspending group support sessions, limiting the length of sessions and notifying parents of every case of suicidal ideation to recommend external professional evaluation. Responding to the needs of the bereaved also prompted a change from protocol when establishing the bereavement support group assessed by Clark et al. (27) whereby the 2 h support meetings were changed from once a month to twice a month. This ensured there was adequate time to provide support to any severely distressed members and provided time for facilitators to debrief and unwind (27). Throughout the process, due to the risk of re-grieving for facilitators in contact with people early in their grief journey, facilitators now rotate each session and support workers provide support for facilitators (27). These changes in protocol speak directly to the fidelity of the program, assessing if the interventions were implemented as originally prescribed.

Interviews with peer support workers who implemented the Peer Support Program (33) identified minor issues with fidelity of implementation. However, these were introduced as concerns to be addressed moving forward, they were not addressed in the life of the evaluation, as above. Feedback included “*frustration if their partner was being unresponsive to their overtures of assistance; uncertainty about what were appropriate boundaries and how to set and maintain them… dissatisfaction with the management of peer matches that were providing unsuccessful coupled with the uncertainty of how to terminate such relationships*” [(33), p. 923]. Despite this, peer supporters and participants offered “*minimal suggestions for improvement*” [(33), p. 923] and peer supporters “*appreciated the format and that ‘there are not a lot of rules to follow’*” [(33), p. 923], indicating overall limited concerns with the protocol of the intervention.

### 3.8 Adoption

Daigle et al. (34) was the only study to explicitly examine program adoption. The Intervention Intensity Checklist and the Intervention Narrative Checklist developed by the authors were employed to observe and rate the adoption of the Group Therapy Program for Children Bereaved by Suicide among therapists (34). The evaluation identified that out of the 13 possible types of intervention, therapists largely relied on just five interventions (34). “*B1 (surround children with respect, authenticity, a presence, attentive listening, empathy) was the activity most observed. Interventions related to conveying knowledge (C1–C3) were less frequent*” [(34), p. 355]. Understanding which of the 13 interventions were most used helped the authors to recommend refining the model of implementation by weighing each of the activities.

### 3.9 Implementation cost, penetration, and sustainability

None of the included studies explicitly evaluated these implementation outcomes. However, Hill et al. (30) briefly commented on concern about the sustainability of the program given it was conditional on the availability of support services and identified the need for “*resourcing to draw everyone else together*” [(30), p. 8] to sustain the program in an already stretched and understaffed environment.

## 4 Discussion

The aim of this scoping review was to examine how suicide postvention programs have been implemented, how implementation has been evaluated, and which implementation outcomes have been evaluated.

One of the key findings of this review was the absence of explicit implementation frameworks used to inform the implementation or evaluation of postvention programs. This was also a common finding in other scoping and systematic reviews investigating implementation in the suicide prevention and broader mental health fields (41–44). Shin et al. (44) identified that limited integration of information and communication technology-based interventions for suicide prevention into clinical practice was “*partly attributable to the lack of theoretical foundations and rigour in research for implementation*” (p. 38). Ellis et al. (42) provided a strong recommendation that future research should incorporate appropriate implementation frameworks, theories or models to guide more structured implementation research. Utilising an implementation framework would help to explore implementation strategies in more detail and provide a more well-rounded understanding of barriers and facilitators to implementation in the field of suicide postvention.

Further, utilising an implementation framework should prompt a consideration of a greater number of implementation outcomes. Proctor et al. (21) recommended exploring each of the outcomes independently to gather a comprehensive view of implementation. Ellis et al. (42) in their review of implementation of e-mental health interventions for depression and anxiety, identified that 70% of the included studies that used an implementation framework assessed three or more implementation outcomes compared to 34% of studies that did not use a framework. This is consistent with our review where 10 out of 16 studies analysed fewer than three implementation outcomes and none of these used specific, predefined conceptual definitions or an implementation framework.

Utilising an implementation framework helps ensure that long-term implementation factors are not overlooked. In their review, Ellis et al. (42) identified that 75% of the included studies that used a framework assessed penetration and 66% assessed sustainability. These outcomes are considered more difficult to investigate given the methodological challenges involved in longer-term follow-up (42). Shin et al. (44) reported that no studies included in their review evaluated the longer-term sustainability of implementation. This aligns with the studies included in our review whereby sustainability was only commented on briefly in one study (30) and neither longer-term outcome was explored in any of the other studies. Ellis et al. (42) also identified fidelity as a mid-stage implementation outcome that was assessed more rarely compared to other outcomes which aligns with the findings of this review (33% assessed fidelity in Ellis et al. (42), 25% of studies assessed fidelity in this review). This may also explain the heavy reliance on assessing feasibility, acceptability, and appropriateness compared to the other outcomes from Proctor et al. (21) framework identified by our review. Shin et al. (44) recommends considering implementation outcomes such as sustainability early in the design process to help drive the successful implementation of interventions over the long-term.

In this review, five studies were identified as using an outcome from Proctor et al. (21) framework such as acceptability or appropriateness without explicitly utilising the framework. This is consistent with other evaluations of mental health program implementation including Palacios et al. (45) and Santucci et al. (46) which both explored feasibility and acceptability, without reference to a theoretical implementation framework. A proxy measure of feasibility was not used by any of the studies in this review aside from a brief discussion of poor retention and recruitment in two studies (23, 25). All other studies discussed components of feasibility from a qualitative perspective. This could be attributed to the lack of formal assessment of feasibility as we mapped this data to feasibility in the framework (21), rather than the authors explicitly aiming to measure feasibility. Lack of consistent terminology and structure in analysis can make it difficult to compare results from different evaluations. Utilising appropriate frameworks will likely lead to greater consistency (42).

Other studies provide insight into how Proctor et al. (21) implementation outcomes can be operationalised. A systematic review by Lattie et al. (47) explored the implementation of digital mental health interventions explicitly using components of Proctor et al. (21) framework. They found that usability and/or acceptability were most commonly assessed using single-item Likert scales, questionnaires or user feedback. This contrasts with the process evaluation by Tsantila et al. (48) which evaluated acceptability and appropriateness using focus group discussions. Both strategies were utilised by studies included in this review. Tsantila et al. (48) used a customised 5-point Likert scale post-intervention survey to measure feasibility which contrasts with the studies in this review which more commonly used qualitative methods to assess feasibility.

Lattie et al. (47) found that studies used number of downloads or uses as measures of adoption while Tsantila et al. (48) used customised monitoring measurements, complimented by focus group discussions exploring experiences with recruitment to analyse adoption. Measures like this are fairly similar to the way in which Daigle et al. (34) evaluated the adoption of interventions most commonly used by therapists. Lattie et al. (47) also used the number of users identified in the adoption section relative to the population of potential users to determine penetration of the intervention. This was not identified by any of the studies included in this review. Fidelity was measured according to study attrition (47); however, retention rates (23, 25) were mapped to feasibility in this review. Neither Lattie et al. (47) or Tsantila et al. (48) explicitly explored the sustainability or implementation cost outcomes. Multiple reviews also noted an absence of discussion of implementation cost in their included studies, which aligns with the findings of this review (41, 42, 44).

These studies (47, 48) highlight that using a particular framework does not limit the way in which implementation outcomes are operationalised, rather they can be utilised in a manner that is suitable for the research question. However, frameworks provide a useful structure to analyse a fuller spectrum of implementation components and help ensure implementation outcomes are considered separately to effectiveness outcomes. Frameworks also offer more consistent terminology and definitions of outcomes which can aid in comparison between studies.

Lack of reliance on validated tools to analyse implementation outcomes was common across the studies included in this review. Although some studies used validated tools such as the Beck Youth Inventories of Emotional and Social Impairment (34, 38), or the Brief Symptoms Ratings Scale-5 (36, 39), they are primarily tools to evaluate effectiveness that were used in the relevant studies as a proxy measure of appropriateness. In their review, Ellis et al. (42) identified a similar absence of validated tools. Metter et al. (49) systematic review identified an increasing number of available valid and reliable measures to measure implementation outcomes. However, they recognised that there is still a need for a coordinated effort to develop high-quality implementation measures (49).

Many of the studies included in this review used Likert-scale questionnaires to assess acceptability by measuring helpfulness, usefulness, benefit and satisfaction. Santucci et al. (46) also sought to evaluate acceptability of the intervention using a quantitative survey and did so with the Client Satisfaction Questionnaire (50). Relying on such validated tools could be a method to increase the rigour of implementation research. It is important to note that not all of the included studies explored their surveys in detail, and they could have relied upon a validated survey or used a researcher-developed survey without clearly identifying it as such. Therefore, it is difficult to draw a precise conclusion about the use of validated tools to assess acceptability and other outcomes of implementation.

Given the notable focus on implementation science within the last 10–20 years, it was surprising that almost 70% (11/16) of studies were published more than ten years ago. Previously, studies mainly focused on the effectiveness of interventions and an explicit focus on implementation was beyond the scope of many studies. The postvention research field should place increased importance on implementation science and continue to publish research on this topic.

### 4.1 Strengths and limitations

A key strength of this review was the variety of study settings that were included. The studies included a range of populations (including children and adults), modes (face-to-face and online), locations and also included perspectives on providers of postvention support.

A limitation of this review was the adoption of a single implementation framework (21). There may have been other suitable frameworks that could have provided other insights into the implementation of postvention programs. Future studies should be conducted in other countries as well.

Due to the limited number of studies included in this review that evaluated implementation strategies from the perspective of providers of postvention support programs, it was not possible to explicitly analyse this unique point of view. Future studies could include the perspectives of providers of support to provide a more well-rounded view of implementation. Future reviews may also include a risk of bias assessment of the included studies.

A key limitation of the studies included in this review was that no study specifically evaluated postvention support for culturally and linguistically diverse populations. Previous research investigating ethnic minority groups’ experiences of suicide bereavement identified greater stigma and shame regarding suicide in some cultural groups, which may hinder the implementation of designated support (51). Therefore, further research on the implementation of suicide postvention programs for culturally and linguistically diverse populations is needed.

## 5 Conclusion

Results from the studies included in this review indicate participants in postvention programs have generally positive feedback alongside recommendations for improvements. Barriers to implementation identified by authors should be carefully considered to improve the implementation of postvention programs in the future.

Recommendations for future research and practice in the postvention field includes aiming to identify and utilise a relevant implementation framework to guide evaluation of postvention programs. This will help provide a well-rounded view of implementation, which explicitly considers a variety of implementation outcomes, encourages attention to longer-term implementation factors, and aids greater conceptual clarity and more consistent terminology. Increasing the use of validated tools to measure implementation will increase the rigour of implementation research. Additionally, suicide postvention is increasingly becoming an integral part of many regional and national suicide preventions strategies and policies, thus applying similar implementation frameworks will increase consistency required for planning and evaluation.

## Data Availability

The original contributions presented in the study are included in the article/Supplementary material, further inquiries can be directed to the corresponding author.
