# Supplementary material for: Implementation of suicide bereavement support: a scoping review
Source: Front Public Health. 2024 Nov 14;12:1474641. doi: 10.3389/fpubh.2024.1474641 (PMC11602391; doi:10.3389/fpubh.2024.1474641)
Supplement: Supplementary file 1 [file Table_1.DOCX]

Supplementary Material – Search String

Search String used in Medline OVID.

1. Suicide/ or suicide*.mp
2. Bereavement/ or bereav*.mp
3. Grief/ or grief.mp or grieving.mp
4. Suicide loss.mp
5. Survivor*.mp
6. Counseling/ or counsel*.mp
7. Intervention.mp
8. (postvention or post-vention).mp or social support/
9. Exp psychotherapy/ or psychotherap*.mp
10. Support group.mp
11. Self-help groups/ or self-help group*.mp
12. Social media/ or social media.mp
13. Internet-based intervention/ or internet/ or internet.mp
14. Outreach.mp
15. Bereavement service*.mp
16. Group survivor meeting*.mp
17. Community response group*.mp
18. Implementation science/ or implement*.mp
19. Knowledge translation.mp
20. Knowledge transfer.mp
21. Knowledge-to-action.mp
22. Improv*.mp
23. Health plan implementation/
24. Guidelines adherence/
25. (Behaviour change or behavior change).mp
26. (Research utilisation or research utilization).mp
27. Guideline*.mp
28. Compliance.mp
29. Adoption.mp
30. Uptake.mo
31. Practice.mp
32. Performance.mp
33. Diffusion.mp
34. Dissemin*.mp
35. Process evaluation.mp
36. Program evaluation.mp or Program evaluation/
37. Delivery.mp
38. Barrier*.mp
39. Facilitator*.mp
40. Enabler*.mp
41. Reach.mp
42. Acceptability.mp
43. Appropriateness.mp
44. Feasibility.mp
45. Satisfaction.mp
46. Sustainability.mp
47. 2 or 3 or 4 or 5
48. 6 or 7 or 8 or 9 or 10 or 11 or 12 or 13 or 14 or 15 or 16 or 17
49. 18 or 19 or 20 or 21 or 22 or 23 or 24 or 25 or 26 or 27 or 28 or 29 or 30 or 31 or 32 or 33 or 34 or 35 or 36 or 37 or 38 or 39 or 40 or 41 or 42 or 43 or 44 or 45 or 46
50. 1 and 47 and 48 and 49

The search string was updated to comply with each of the databases. All of the key terms were used in each database search; however, the MeSH terms were not available for all databases.
